# Deep Convolutional Neural Network-Based Positron Emission Tomography Analysis Predicts Esophageal Cancer Outcome

**DOI:** 10.3390/jcm8060844

**Published:** 2019-06-13

**Authors:** Cheng-Kun Yang, Joe Chao-Yuan Yeh, Wei-Hsiang Yu, Ling-I. Chien, Ko-Han Lin, Wen-Sheng Huang, Po-Kuei Hsu

**Affiliations:** 1 aetherAI, Co., Ltd., Taipei 112, Taiwan; jimmy15923@gmail.com (C.-K.Y.); joeyeh@dysklabs.com (J.C.-Y.Y.); vashineyu@gmail.com (W.-H.Y.); 2 Department of Nursing, Taipei Veterans General Hospital, Taipei 112, Taiwan; Lichien@vghtpe.gov.tw; 3 Department of Nuclear Medicine, Taipei Veterans General Hospital, Taipei 112, Taiwan; khlin3@vghtpe.gov.tw (K.-H.L.); wshuang2@vghtpe.gov.tw (W.-S.H.); 4 Division of Thoracic Surgery, Department of Surgery, Taipei Veterans General Hospital and School of Medicine, National Yang-Ming University, Taipei 112, Taiwan

**Keywords:** deep convolutional neural network, esophageal cancer, PET

## Abstract

In esophageal cancer, few prediction tools can be confidently used in current clinical practice. We developed a deep convolutional neural network (CNN) with 798 positron emission tomography (PET) scans of esophageal squamous cell carcinoma and 309 PET scans of stage I lung cancer. In the first stage, we pretrained a 3D-CNN with all PET scans for a task to classify the scans into esophageal cancer or lung cancer. Overall, 548 of 798 PET scans of esophageal cancer patients were included in the second stage with an aim to classify patients who expired within or survived more than one year after diagnosis. The area under the receiver operating characteristic curve (AUC) was used to evaluate model performance. In the pretrain model, the deep CNN attained an AUC of 0.738 in identifying patients who expired within one year after diagnosis. In the survival analysis, patients who were predicted to be expired but were alive at one year after diagnosis had a 5-year survival rate of 32.6%, which was significantly worse than the 5-year survival rate of the patients who were predicted to survive and were alive at one year after diagnosis (50.5%, *p* < 0.001). These results suggest that the prediction model could identify tumors with more aggressive behavior. In the multivariable analysis, the prediction result remained an independent prognostic factor (hazard ratio: 2.830; 95% confidence interval: 2.252–3.555, *p* < 0.001). We conclude that a 3D-CNN can be trained with PET image datasets to predict esophageal cancer outcome with acceptable accuracy.

## 1. Introduction

Accurate estimates of survival are of paramount importance for patients and oncologists making personalized and patient-centered decisions in the era of precision medicine. Many clinical prediction tools combining key prognostic factors have been proposed to improve risk stratification and prognostication in esophageal cancer. However, few existing tools can be confidently used in current clinical practice [1]. Besides clinical parameters, medical image analysis is also gaining exponential interest in prognostic research. It has been proposed that radiomics analysis, which is defined as the high-throughput extraction of quantitative image metrics, potentially facilitates characterization of tumor phenotypes and even predicts patient survival [2,3,4]. Several studies have developed radiomics prognostic models incorporating positron emission tomography (PET) image features, which contain both anatomic and metabolic information, in lung cancer, colorectal cancer, and esophageal cancer [3,4,5,6]. Recently, the mainstream of computational image analysis has been gradually replaced by deep learning techniques [7,8,9]. The most impactful feature of the deep learning algorithm is self-learning; once a data set has been provided, the program can automatically discover features and adaptively learn without human indication by a backpropagation algorithm and by changing the internal parameters of each layer [7,8]. Deep learning algorithms, in particular convolutional neural networks (CNNs), have been applied in medical image classification, segmentation, and detection [8,9], e.g., skin lesions classification [10], diabetic retinopathy detection [11], and diagnosis of lymph node metastasis in breast cancer [12]. Pilot studies even demonstrated the potential of CNNs to recognize biological features that are overlooked by human experts and predict someone’s risk for certain diseases [13,14]. However, the application of deep learning in cancer survival prediction is limited in the literature. We hypothesize that a deep learning algorithm facilitates PET image analysis in predicting esophageal cancer outcome. In the present study, we trained a CNN to identify esophageal cancer patients with extremely poor prognosis, i.e., expired within one year after diagnosis, using only pretreatment PET images.

## 2. Methods

### 2.1. Data Source

The PET scans of patients with the diagnosis of esophageal squamous cell carcinoma between September 2009 and August 2017 at Taipei Veterans General Hospital were collected. The PET scans of patients with stage I lung cancer diagnosed between January 2012 and November 2017 at Taipei Veterans General Hospital were also collected for use in pretraining the CNN. To focus on critical information, only PET images from the hypopharynx to the stomach, which included all of the esophagus and the peri-esophageal regions were used.

### 2.2. Convolution Neural Network Model Setup and Training

Input images were classified as positive if the patient expired within a year after diagnosis, while those corresponding to patients that survived more than one year after diagnosis were classified as negative. We built a three-dimensional (3D) CNN based on a residual network, ResNet [15], as illustrated in Figure S1. To achieve better performance and arrive at convergence (that is, optimum performance) faster, we used PET scans from patients with stage I lung cancer, whose esophagi were presumed to be normal, and trained the network as our first stage of training. We used the PET scans to pretrain our model with an aim to classify the scans into esophageal cancer (abnormal esophagus) or lung cancer (normal esophagus). For the second stage, which aimed to classify esophageal cancer outcome, model weights were transferred from the aforementioned pretrained model. We used an 18-layer ResNet with the stochastic gradient descent (SGD) optimizer and anisotropic max-pooling as the baseline model and compared different model structures/hyper-parameters. For each experimental setting, one of the components was modified and compared with the baseline model. The experimental conditions included: (1) with or without pretraining; (2) choice of optimizer (adaptive momentum estimation (Adam) vs. SGD); and (3) number of layers (18- vs. 34-layer residual network). The final model contained 34 layers with a bottleneck design containing about 33 million learnable parameters. The detailed data preprocessing, model setup, and hyper-parameter settings are shown in supplementary files.

### 2.3. Statistics

We randomly shuffled the data index before splitting the entire dataset into five subgroups of equal size. We repeated the process of model training using one subgroup as a validation set and the rest as a training set until all subgroups were used. With each subgroup as a validation set, we trained the deep neural network in five independent runs and used the average area under receiver-operating characteristic (ROC) curve (AUC) to evaluate its performance, as shown in Figure S2. Based on the probability threshold of 0.5, patients were predicted to expire within one year after diagnosis (positive) or not (negative). The prediction results were compared to clinical data and patients were classified into correct or incorrect prediction groups. The correct prediction group included patients whose survival status was the same as predicted status; whereas those who were predicted to be expired (or alive) but were alive (or expired) at one year were classified in the incorrect prediction group. Overall survival (OS) was defined as the time from the date of diagnosis until death or last known follow-up. Survival curves were plotted using the Kaplan–Meier method and compared by the log-rank test. Multivariable survival analysis, which included all available clinical factors, was conducted with a Cox regression model. Survival analysis was performed using Statistical Package for the Social Sciences (version 17, SPSS Inc., Chicago, IL, USA) and a two-sided *p*-value < 0.05 was considered significant.

## 3. Results

### 3.1. Patients

A total of 798 PET scans of esophageal squamous cell carcinoma and 309 PET scans of stage I lung cancer were included in the pretraining stage of this study. In the esophageal cancer outcome classification stage, only the pretreatment PET scans were used and patients without complete clinical follow-up data were excluded. The characteristics of final 548 patients are shown in Table 1.

### 3.2. CNN Performance

**Figure 1:**
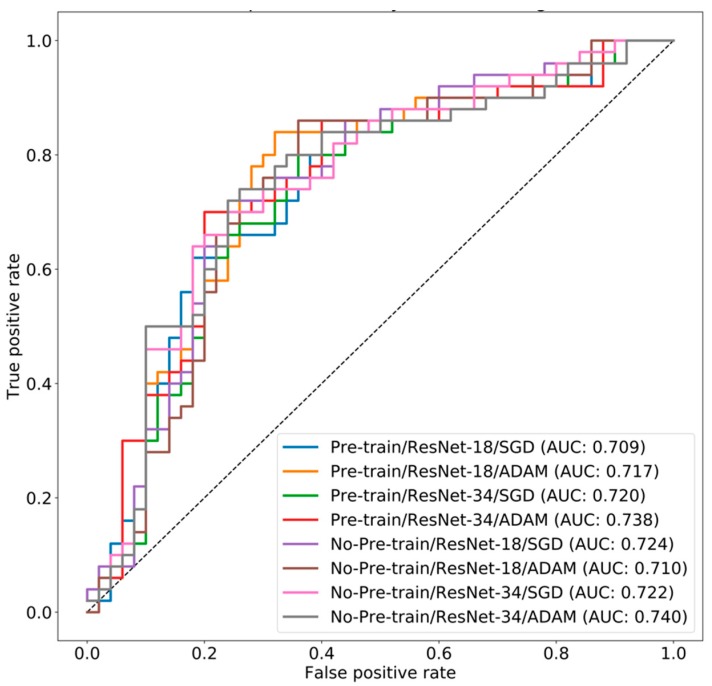
Receiver-operating characteristic curves of different CNN models.

The performance of each model is shown in Table 2, and distribution of AUC for each experiment is shown in Figure S3. The ROC curve comparison between different hyper-parameters and structure combinations is illustrated in Figure 1. In general, there was no difference between models with pretraining and models without pretraining. The model performances were comparable between the SGD optimizer and the Adam optimizer. Finally, we compared the 18-layer residual network and the 34-layer residual network. In the pretrained models, the 34-layer network achieved better results than the 18-layer network without over-fitting (0.738 AUC vs. 0.717 AUC with the Adam optimizer and 0.720 AUC vs. 0.709 AUC with the SGD optimizer). In the non-pretrained models, the 34-layer network had better results than the 18-layer network with the Adam optimizer (0.740 AUC vs. 0.710 AUC) and had similar results with the SGD optimizer (0.722 AUC vs. 0.724 AUC). 

### 3.3. Clinical Relevance

The comparison between patients with correct and incorrect prediction (according to the pretrained model of 34-layer network with the Adam optimizer) is shown in Table 1. These two groups were similar with regard to age, gender, clinical T stage, clinical M stage, tumor location, tumor size, maximal standard uptake value (SUVmax), value of tumor on PET scan, serum level of tumor markers, and treatment modalities. The only difference is the clinical N stage. The incorrect prediction group had more clinical N1 patients than the correct prediction group (46.3% vs. 31.9%, *p* = 0.016). 

**Figure 2:**
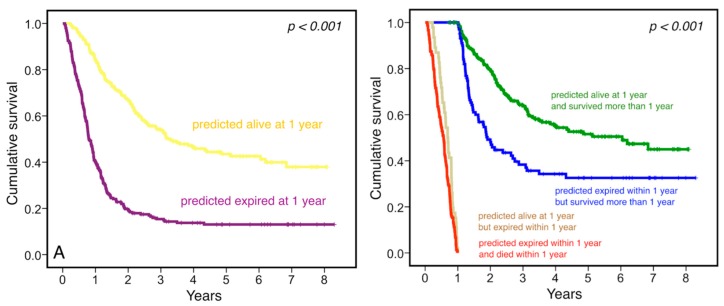
Prognostic significance of 3D-CNN prediction results. **A**: yellow: patients predicted alive at one year; purple: patients predicted expired at one year. **B**: green: patients predicted alive at one year and survived more than one year after diagnosis; blue: patients predicted expired within one year but survived more than one year; tan: patients predicted alive at one year but expired within one year; red: patients predicted expired and died within one year, *p* < 0.001.

To investigate the prognostic impact of prediction results, survival analysis was performed based on prediction results and clinical status, as shown in Figure 2. Patients who were predicted to survive and survived one year after diagnosis had the highest 5-year survival rate of 50.5%. Interestingly, patients who were predicted to be expired but survived at one year after diagnosis had a 5-year survival rate of 32.6%, with a median survival of 23.2 (95% CI: 19.3–27.1) months, which was significantly worse than the outcome of the patients who were predicted to survive and really survived one year after diagnosis (*p* < 0.001). In the multivariable survival analysis, the independent prognostic factors for overall survival included age, gender, tumor location at upper third, clinical N stage, clinical M stage, and the prediction results by our CNN model, as shown in Table 3 (hazard ratio (HR): 2.830; 95% confidence interval (CI): 2.252–3.555, *p* < 0.001). 

## 4. Discussion

Outcome prediction in cancer care is the foundation for individualized treatment planning. Prediction tools integrating cancer stage, age, sex, comorbidities, treatment received, treatment response, and specific surgical pathology have been developed for esophageal cancer prognostication. However, few existing survival prediction tools are ideal for use in current clinical practice. In a review by Gupta et al., the discrimination ability, which might vary from 0.5 (model predictions are similar to chance) to 1.0 (model predictions are perfect), of sixteen clinical tool-based models in the literature was around 0.63 to 0.77, which was far from confident application in clinical use [1]. In addition to patient specific clinicopathological information, medical image analysis is gaining substantial interest in prognostic research. In oncology, the SUV of 18-fluorodeoxyglucose (FDG), which measures FDG activity in the tumor and correlates with viable tumor cell number and metabolism, obtained from FDG-PET scans is widely used for diagnosing, staging, monitoring response to therapy, as well as outcome prediction [16,17,18]. Correlation between higher SUVmax and worse survival has been reported in several studies [19,20,21]. Owing to non-uniformly standardized PET imaging protocols, variable SUVmax thresholds have been reported and thus limit its use [22]. Besides conventional uptake parameters, texture analysis, which provides numerous quantitative and semiquantitative indices, termed “features” [2,3,4], seems to perform better in characterizing tumor phenotypes [23]. This approach as a whole is named “radiomics” [24]. Several studies have successfully developed radiomic prognostic classifiers that can be associated with metastatic recurrence and survival in several types of cancers [3,4,16]. For example, Lambin et al. demonstrated that the prognostic model based on pretreatment CT radiomic features yielded an AUC of 0.69 in predicting 3-year overall survival of esophageal cancer patients after chemoradiotherapy [25]. Although many different PET segmentation techniques have been proposed and numerous PET-based radiomic features have been described, the results are highly dependent on the method used [4,16,26]. For example, in an external validation study of a prognostic model incorporating quantitative PET image features in esophageal cancer, Foley et al. has shown that results of a developed prognostic model combining clinical factors and PET radiomic features was not replicated in another cohort of patients treated with different regimens [26]. Another limitation in radiomics research is the algorithm that extracts imaging features through manual detection and characterization of tumor regions, which is labor intensive and subjective. In contrast, deep learning algorithms, in particular convolutional neural networks, provide workflows that allow automated selection and quantification of the most robust features. Since tumor segmentation or feature calculation is not required, it simplifies the analysis procedure and is more objective than the classical methods [7,8,9,27].

The application of CNNs in clinical medical imaging is on the horizon. The performance of CNNs can be outstanding with an abundance of well-annotated data; for example, classification of skin cancer [10], classification of dermoscopic melanoma recognition [28], detection of diabetic retinopathy with retinal fundus photographs [11], detection of lymph node metastases with whole slide images in breast cancer [12], and anatomical classification of esophagogastroduodenoscopy images [29]. Intriguingly, it has been proposed that CNNs may be able to astutely reveal subtle biological characteristics that are not visible to physicians. As examples, researchers have used CNNs to predict cardiovascular risk factors with retinal fundus photographs [14]. In thoracic oncology, a CNN has been trained to classify mediastinal lymph nodes of non-small cell lung cancers with FDG-PET images [30]. In another study, Ypsilantis et al. used PET imaging data from 107 patients with esophageal cancer to demonstrate that a CNN has the potential to predict chemotherapy response [31]. Applying deep learning networks in predicting overall survival, Hosny et al. trained a CNN from CT data of lung cancer patients treated with radiotherapy or surgery. The prognostic power attained an AUC of 0.70 and they concluded that the CNN is capable to significantly stratify patients into low and high mortality risk groups [27]. 

In this study, we trained a CNN to predict the survival status at one year after diagnosis in esophageal cancer patients. Our model attained an AUC of 0.738, which was better than that of clinical factor-based prediction models, as shown in Figure S4. The AUC of our model was also better than that of a CT radiomic feature-based model in predicting survival status in esophageal cancer (AUC: 0.69 [25]), and that of a deep learning-based model in predicting mortality risk in lung cancer (AUC: 0.70 [27]). In the multivariable analysis, the prediction result was an independent prognostic factor, indicating that our model could identify esophageal cancer with aggressive behavior. Interestingly, when we compared correct and incorrect prediction groups, the only difference was the percentage of clinical N1 stage. Indeed, the outcome of patients with minimal nodal involvement was highly dependent on either the extent of surgical resection or response to systemic treatments, which could not be revealed by pretreatment PET scan images. 

To overcome the limitation of a relatively small dataset, we adopted a stepwise workflow, in which the first stage was to classify normal and abnormal esophagi only. Another limitation is the nature of opaqueness of deep learning networks. Feature definition, extraction, and selection are all automated and occur implicitly. Consequently, the imaging characteristics they measure are highly obscure. This ambiguity is in sharp contrast to the expert-based well-defined radiomic features. For example, it has been proposed that morphometric measures, such as core muscle size, obtained from cross-sectional imaging, was correlated with sarcopenia, which is an independent predictor of clinical outcomes in multiple gastrointestinal cancers [32,33]. The CNN classification may be based on tumor or non-tumor features. Moreover, we did not include PET images from different PET scanners. Further studies are required to test the reliability and robustness of our results. Lastly, our proposed model was not compared against other machine learning-based methods. In the future, it is mandatory to apply other methods on our dataset and compare the AUC performance.

In conclusion, a 3D-CNN can be trained with PET image datasets to predict outcome in esophageal cancer with acceptable accuracy. The prediction result remained an independent prognostic factor in multivariable survival analysis. Although our current results cannot be readily applied to clinical decision making, we demonstrated the potential of deep learning. With a larger dataset, the CNN can be trained to achieve a better prediction performance.

## Figures and Tables

**Table 1:** Patient demographics.

	Total	Correct Prediction	Incorrect Prediction	*p*
	*n* = 548	*n* = 401	*n* = 147	
Age, (years, mean ± SD)	61.4 ± 12.4	61.7 ± 12.7	60.5 ± 11.7	0.290
Gender (%)				0.320
Male	504 (92.0)	366 (91.3)	138 (93.9)	
Female	44 (8.0)	35 (8.7)	9 (6.1)	
Clinical T stage (%)				0.109
T1	65 (11.9)	55 (13.7)	10 (6.8)	
T2	118 (21.5)	89 (22.2)	29 (19.7)	
T3	325 (59.3)	229 (57.1)	96 (65.3)	
T4	40 (7.3)	28 (7.0)	12 (8.2)	
Clinical N stage (%)				0.016
N0	178 (32.5)	141 (35.2)	37 (25.2)	
N1	196 (35.8)	128 (31.9)	68 (46.3)	
N2	102 (18.6)	76 (19.0)	26 (17.7)	
N3	72 (13.1)	56 (14.0)	16 (10.9)	
Clinical M stage (%)				0.970
M0	458 (83.6)	335 (83.5)	123 (83.7)	
M1	90 (16.4)	66 (16.5)	24 (16.3)	
Tumor location (%)				0.349
Upper third	161 (29.4)	111 (27.7)	50 (34.0)	
Middle third	242 (44.2)	182 (45.4)	60 (40.8)	
Lower third	145 (26.5)	108 (26.9)	37 (25.2)	
Tumor size, (cm, mean ± SD)	4.4 ± 3.4	4.3 ± 3.3	4.5 ± 3.5	0.730
SUV_MAX_, (mean ± SD)	12.5 ± 6.0	12.3 ± 6.2	12.9 ± 5.4	0.308
Tumor markers, (mean ± SD)				
SCC, ng/ml	2.6 ± 5.3	2.6 ± 6.0	2.6 ± 2.8	0.987
Cyfra 21-1, ng/ml	4.2 ± 5.9	4.4 ± 7.2	4.1 ± 5.9	0.879
Treatments (%)				0.758
Primary resection	141 (25.7)	101 (25.2)	40 (27.2)	
Trimodal treatments	160 (29.2)	115 (28.7)	45 (30.6)	
Medical treatment	208 (38.0)	154 (38.4)	54 (36.7)	
Supportive care	39 (7.1)	31 (7.7)	8 (5.4)	

SD: standard deviation; SUV_MAX_: maximal standard uptake value; SCC: squamous cell carcinoma antigen. Data were available in 470 (size), 489 (SUV), 387 (SCC), and 139 (Cyfra 21-1) patients owning to respective nature of this study.

**Table 2:** Performance of CNN models.

Models			AUC	95% CI
Pretrain	Optimizer	Layers		
No	Adam	18	0.710	0.688–0.733
No	SGD	18	0.724	0.714–0.734
No	Adam	34	0.740	0.704–0.776
No	SGD	34	0.722	0.697–0.747
Yes	Adam	18	0.717	0.693–0.741
Yes	SGD	18	0.709	0.678–0.741
Yes	Adam	34	0.738	0.714–0.761
Yes	SGD	34	0.720	0.694–0.746

CNN: convolutional neural network, AUC: area under curve; CI: confidence interval.

**Table 3:** Multivariable overall survival analysis.

	HR	95% CI	*p*
Age	1.018	1.010–1.027	<0.001
Gender			
Male	1	-	-
Female	0.426	0.252–0.720	0.001
Tumor location			
Lower third	1	-	-
Middle third	1.297	0.960–1.752	0.090
Upper third	1.333	1.007–1.763	0.044
Clinical T stage			
T1/2	1	-	-
T3/4	1.209	0.946–1.545	0.130
Clinical N stage			
N(−)	1	-	-
N(+)	1.417	1.082–1.856	0.011
Clinical M stage			
M0	1	-	-
M1	2.041	1.571–2.651	<0.001
Prediction results			
Alive at one year	1	-	-
Expired within one year	2.830	2.252–3.555	<0.001

HR: hazard ratio; CI: confidence interval.

## Supplementary Materials

The following are available online at https://www.mdpi.com/2077-0383/8/6/844/s1, Figure S1: 3D residual network overview, Figure S2: Process of data training and validation, Figure S3: Scatter plot demonstrating the distribution of AUC for each experiment, Figure S4: ROC curves of clinical factor-based models in predicting the survival status at one year after diagnosis.
