# Supplementary material for: Deep Convolutional Neural Network-Based Positron Emission Tomography Analysis Predicts Esophageal Cancer Outcome
Source: J Clin Med. 2019 Jun 13;8(6):844. doi: 10.3390/jcm8060844 (PMC6616908; doi:10.3390/jcm8060844)
Supplement: Supplementary file 1 [file jcm-08-00844-s001.pdf]

Supplement

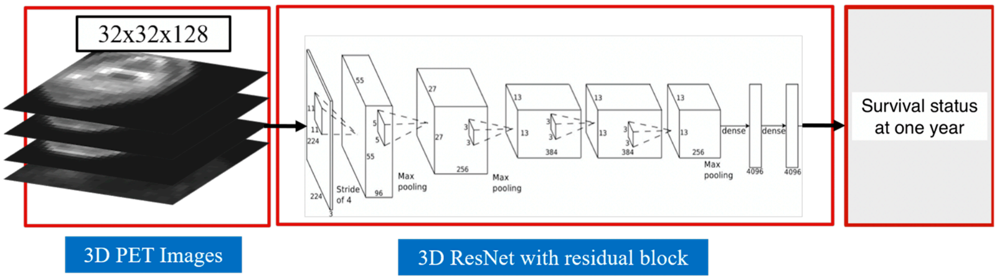

Figure S1. 3D residual network overview.

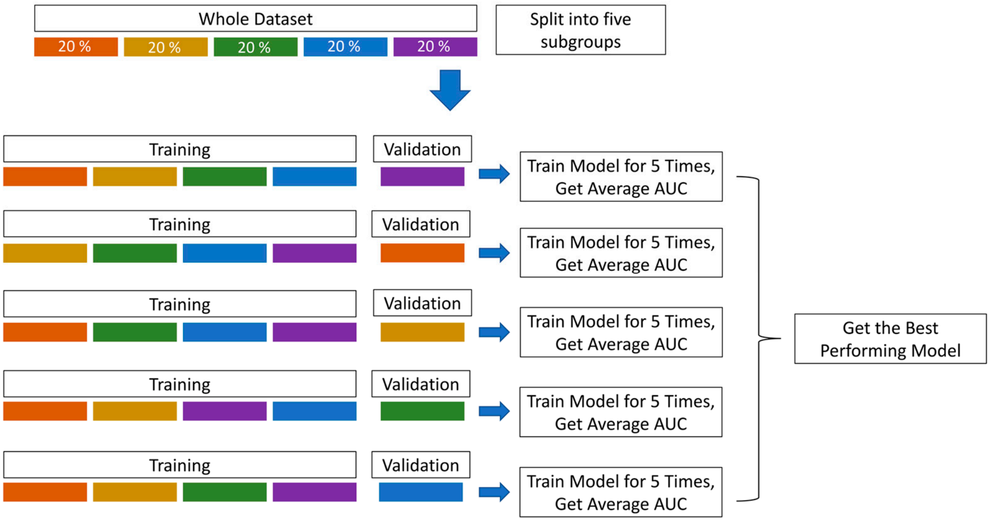

Figure S2. Process of data training and validation.

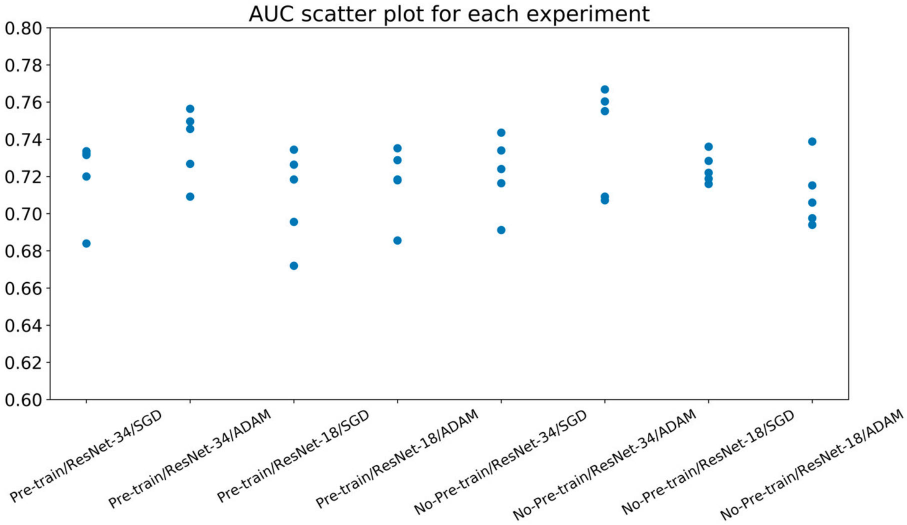

Figure S3. Scatter plot demonstrating the distribution of AUC for each experiment.

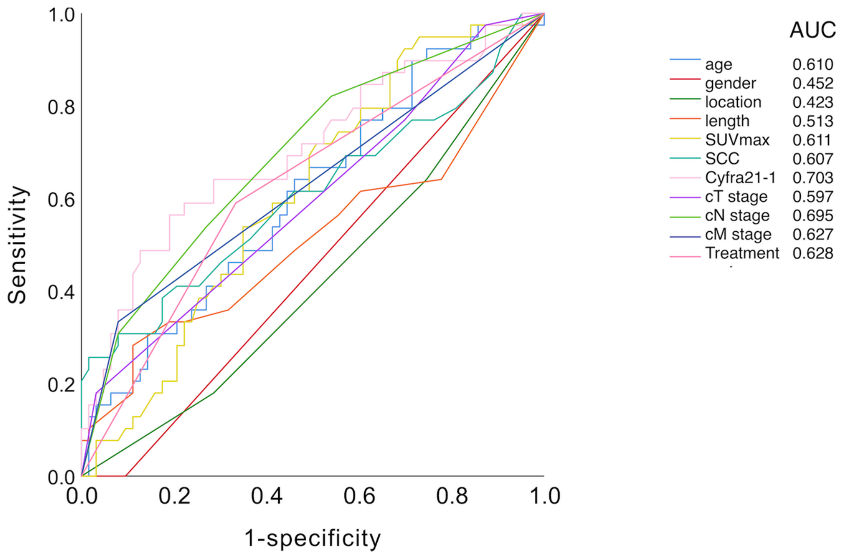

Figure S4. ROC curves of clinical factor based models in predicting the survival status at one year after diagnosis.

Convolution neural network model setup and training

Data source and preprocessing

The pixel resolution of each PET scan in XY was 128 x 128, with a pixel size of 5.47 x 5.47 mm and a slice thickness of 3.27 mm. Raw PET images in DICOM format were first converted to standardized uptake value images. To focus on critical information, we cropped images to remove any irrelevant areas. Each cropped PET scan covered the body area from the hypopharynx to the stomach and included all of the esophagus and the peri-esophageal regions. The field of view of each cropped scan was 32 x 32 x 128 pixels. Additionally, we increased the effective size of the training set using on-the-fly data augmentation. Six augmentation methods were employed in this study: random image translation, random scaling, random rotation, random left/right flipping, random swapping of anterior/posterior view, and random Gaussian blurring. Finally, each input image was zero-centered by subtracting the mean and the min/max normalized by dividing the image by its intensity range.

Model setup

We built a three-dimensional (3D) convolution neural network (CNN) based on a residual network, ResNet. For our neural network, we applied full pre-activation, which reorganizes the order of convolutions and activation functions so that batch normalization and a rectified linear unit (ReLU) precede convolution layer. This results in better performance compared to original residual block or other configurations, such as ReLU before addition or ReLU-only pre-activation[1]. Because the PET scans used in this study did not have isotropic spatial resolution in X, Y, and Z, we used an anisotropic max-pooling layer following the first convolution layer to compensate for this fact as well as to reduce the model size. After several convolutions, we used a global averaging method to flatten the extracted features as a vector. This vector was then connected to the dense layer using a softmax function, with the output being the probability of a given image being classified and predicting the expired time after diagnosis.

Hyper-parameters setting

In the pretraining stage, the kernel weights of the network were initialized using a recipe published by Kaiming He et al.[2] To train the model, we used Stochastic Gradient Descent (SGD)[3-5] with Nesterov momentum[6,7] (with an initial learning rate of 0.0001 and momentum of 0.9). The batch size was set to 16, and the model was trained on a single nVIDIA TESLA P40 graphics processing unit (GPU). Since the model would over-fit easily if it was trained on an unbalanced dataset, we used a batch balancing method, which mixes over-sampling and under-sampling to circumvent class imbalance in the dataset.[8] This method allowed us to train the model with balanced samples (8 positive and 8 negative samples per batch). To further prevent model over-fitting, reduce-learning-rate-on-plateau and early-stopping were included during the training process. The reduce-learning-rate-on-plateau schema generally dropped the learning rate by half when a validation performance did not improve for a few epochs, and early-stopping caused the model to stop training when a validation performance did not show improvement after 25 epochs (or 750 iterations).

References

1. He K, Zhang X, Ren S, Sun J. Identity mappings in deep residual networks. July 2016. https://arxiv.org/pdf/1603.05027.pdf. Accessed August 3, 2018.
2. He K, Zhang X, Ren S, Sun J. Delving deep into rectifiers: surpassing human-level performance on imagenet classification. February 2015. https://arxiv.org/pdf/1502.01852.pdf. Accessed August 3, 2018.
3. Robbins H, Monro S. A stochastic approximation method. Ann Math Statist. 1951;22(3):400-407.
4. Kiefer J, Wolfowitz J. Stochastic estimation of the maximum of a regression function. Ann Math Statist. 1952;23(3):462-466.
5. Bottou L, Curtis FE, Nocedal J. Optimization methods for large-scale machine learning. February 2018. https://arxiv.org/pdf/1606.04838.pdf. Accessed August 3, 2018.
6. Nesterov YE. A method for unconstrained convex minimization problem with the rate of convergence o(1/k2). Dokl Akad Nauk SSSR. 1983;269:543-547.
7. Sutskever I, Martens J, Dahl G, Hinton G. On the importance of initialization and momentum in deep learning. Proceedings of the 30th International Conference on Machine Learning, PMLR. 2013;28(3):1139-1147.
8. Hernandez J., Carrasco-Ochoa J.A., Martínez-Trinidad JF. An empirical study of oversampling and undersampling for instance selection methods on imbalance datasets. In: Ruiz-Shulcloper J, Sanniti di Baja G, eds. Progress in Pattern Recognition, Image Analysis, Computer Vision, and Applications. New York, NY: Springer; 2013.
